# Inactivation of the *WNT5A* Alternative Promoter B Is Associated with DNA Methylation and Histone Modification in Osteosarcoma Cell Lines U2OS and SaOS-2

**DOI:** 10.1371/journal.pone.0151392

**Published:** 2016-03-15

**Authors:** Himani Vaidya, Candie Rumph, Karen S. Katula

**Affiliations:** 1 Fels Institute for Cancer Research and Molecular Biology, Temple University School of Medicine, Philadelphia, Pennsylvania, United States of America; 2 Department of Biology, The University of North Carolina Greensboro, Greensboro, North Carolina, United States of America; University of Navarra, SPAIN

## Abstract

*WNT5A* is a secreted ligand involved in Wnt pathway signaling and has a role in cell movement and differentiation. Altered *WNT5A* expression is associated with various cancers, although in most studies the focus has been on only one of the known *WNT5A* isoforms. In this study, we analyzed expression from two of the major *WNT5A* promoters, termed promoter A and promoter B, in normal human osteoblasts, SaOS-2 and U2OS osteosarcoma cell lines, and osteosarcoma tumor tissue. We found that both promoters A and B are active in normal osteoblasts with nearly 11-fold more promoter B than A transcripts. Promoter B but not promoter A transcripts are decreased or nearly undetectable in the SaOS-2 and U2OS cell lines and osteosarcoma tumor tissues. Transient transfection of promoter A and promoter B reporter constructs confirmed that SaOS-2 cells have the necessary factors to transcribe both promoters. Bisulfite sequencing analysis revealed that three CpG enriched regions upstream of the promoter B exon 1βare highly methylated in both SaOS-2 and U2OS cells. The CpG island sub-region R6 located in promoter B exon 1β was approximately 51% methylated in SaOS-2 and 25% methylated in U2OS. Region 3 was approximately 28% methylated in normal osteoblasts, whereas the others were unmethylated. Promoter B was re-activated by treatment of SaOS-2 cells with 1 μM 5-azacytidine, which was associated with only a small insignificant change in methylation of sub-region R6. ChIP analysis of U2OS and SaOS-2 cells indicated that the promoter B region is less enriched in the active histone mark H3K4me3, in comparison to promoter A and that there is increased enrichment of the repressive mark H3K27me3 in association with the promoter B genomic region in the cell line SaOS-2. These findings show that epigenetic inactivation of the *WNT5A* promoter B involves both DNA methylation and histone modifications and suggest that differential expression of the *WNT5A* alternative promoters A and B is a characteristic of osteosarcomas.

## Introduction

WNT5A is a secreted glycoprotein that binds Frizzled (Fz) and Receptor tyrosine kinase-like orphan receptor 1/2 (Ror 1/2) receptors, leading to either inhibition or activation of Wnt signaling pathways, depending on the receptor-context of the cell [1, 2]. WNT5A is involved in regulating cell movement and differentiation, particularly of mesenchymal stem cells such as chrondrocytes, adipocytes, and osteoblasts, through activation of the non canonical Wnt RhoA/Rac or calcium (Ca^2+^) signaling pathways [2].

WNT5A is misregulated in a wide range of cancer types, displaying both increased and decreased expression. Cancers in which WNT5A is typically upregulated include melanoma [3], gastric [4, 5, 6], skin [7], pancreatic [8, 9, 10], and osteosarcoma [11, 12]. WNT5A is often downregulated in leukemia [13, 14, 15], colorectal [16, 17], and esophageal [18] cancers. Breast and prostate cancers are more variable showing both increased [19, 20, 21, 22] and decreased WNT5A expression [22, 23, 24]. Of particular importance to this study is the finding that the *WNT5A* gene is silenced by epigenetic mechanisms, primarily DNA methylation but also histone modifications, in cancers in which the gene is not expressed [14, 15, 16, 17, 18, 25, 26].

WNT5A would be expected to display oncogenic functions when upregulated and in various studies, WNT5A overexpression was found to increase proliferation, migration and/or invasion [8, 12, 27, 28]. WNT5A appears to functions as a tumor suppressor when downregulated. As an example, transfection of a WNT5A expression vector into the thyroid tumor cell line FTC-133 led to a *decrease* in proliferation, invasion, and migration [29]. WNT5A was shown to inhibit migration of the SW480 colorectal cancer cell line, which lacks WNT5A [16]. And, loss of WNT5A in hepatocellular carcinoma was associated with poor prognosis [30]. Apparently, WNT5A plays distinct functions in different tumor types, at different tumor stages, and likely in tumors with particular molecular characteristics.

**Fig 1:**
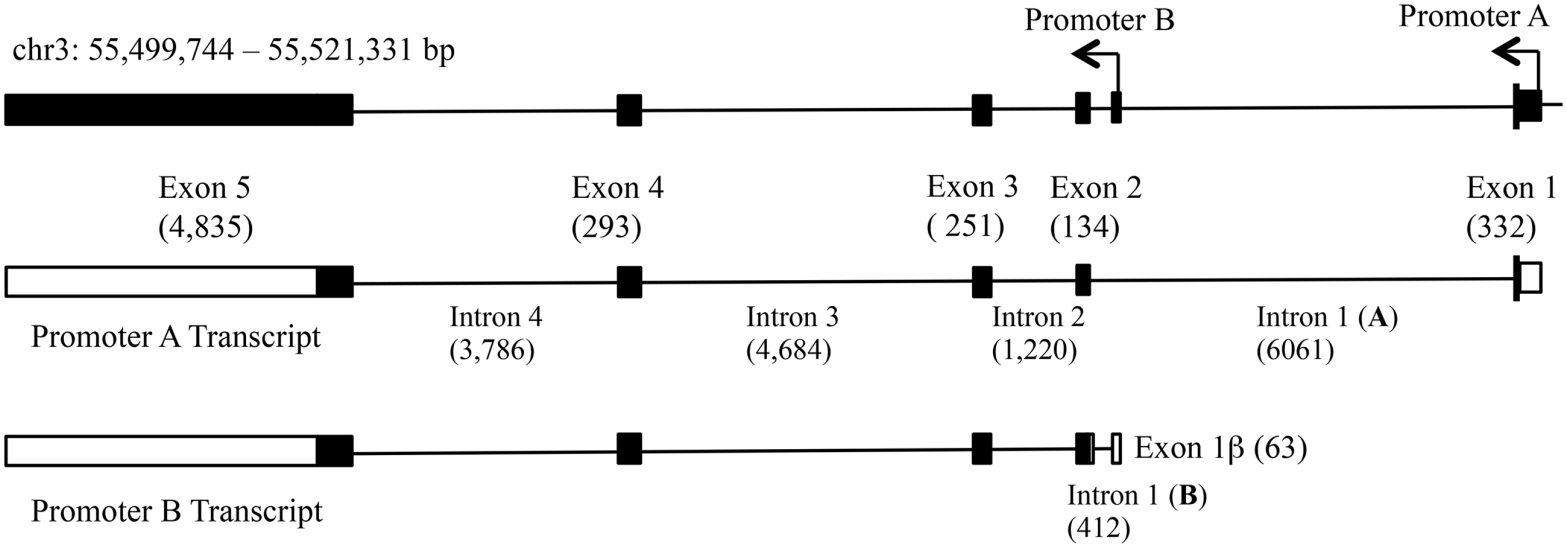
Human *WNT5A* genomic and primary transcripts. Shown is the genomic structure and two of the coding transcripts generated from the *WNT5A* gene region on chr3. The top diagram shows the relative locations of the *WNT5A* promoter A and promoter B starts of transcription (arrows). The black boxes are exon sequences. Shown below this are the derived promoter A and promoter B primary transcripts. Promoter A has a unique exon 1. Promoter B generates a primary transcript with a unique exon 1β and intron 1 (B). Exons 2, 3, 4, and 5 are common to both transcripts. Black boxes are coding exon sequences; open boxes are non coding exon sequences. The numbers in parentheses are sizes in base pair. The diagram was modified from Ensembl *WNT5A* ENSG00000114251 and Bauer et al. [31].

The varied behaviors of WNT5A could also be explained by the unique activities of the WNT5A protein isoforms. The *WNT5A* gene region generates multiple transcripts by alterative splicing and distinct transcription start sites (see Ensembl *WNT5A* ENSG00000114251). Currently, there are eight known human WNT5A transcripts of which three give rise to complete proteins. One of these transcripts and its derived protein isoform has been the focus of nearly all the published studies on WNT5A. This transcript is associated with the cDNA NM_003392. We refer to this transcription unit as the promoter A transcript. Our group has been studying another of the *WNT5A* transcripts, referred to as promoter B. The sequences of the promoter A and promoter B primary transcripts have recently been clarified [31]. The genomic relationship of promoters A and B and their primary transcripts are shown in Fig 1. In a previous study, we showed that the separated promoters A and B are expressed at high levels when transiently expressed in fibroblast cells NIH3T3 and colon epithelial cells Caco-2 and that the promoters are differentially affected by TNF-alpha [32].

The unprocessed protein derived from the promoter A transcript has 15 additional amino acids on its N-terminus in comparison to the promoter B derived protein isoform. Bauer et al. [31] showed that after processing in the rough endoplasmic reticulum, the promoter A derived protein (termed isoform WNT5A-L) differs from isoform B (termed WNT5A-S) by 18 N-terminal amino acids. Functional analyses indicated that isoform WNT5A-S, at least in some cancer cell lines, increases cell proliferation, whereas isoform WNT5A-L reduces proliferation [31]. These data, in combination with our findings, suggest that cell proliferation could be affected by changes in the relative amounts of the WNT5A isoforms that results from differential activities of the A and B promoters.

WNT5A expression was found to increase in some osteosarcomas and to promote migration and invasion of this cancer type [12, 29, 33, 34]. For these studies, however, only the *WNT5A* promoter A derived transcript and the WNT5A isoform WNT5A-L was analyzed. Nothing is known regarding the activities of the *WNT5A* promoters A and B in osteosarcoma cancers. To answer this question, we quantified *WNT5A* promoter A and promoter B specific transcript levels in normal osteoblasts, osteosarcoma cell lines, and primary osteosarcoma tumor tissue. Surprisingly, we found that promoter B transcripts are reduced in both osteosarcoma cell lines and primary tumor tissues. In normal human osteoblasts, there are more promoter B than promoter A transcripts. We determined that inactivation of promoter B in the osteosarcoma cell lines involves DNA methylation but also includes histone modifications, supporting the conclusion that the *WNT5A* alternative promoters are *differentially* regulated by epigenetic mechanisms in osteosarcoma tumors.

## Materials and Methods

### Cell lines, osteosarcoma tumor tissues, and normal human osteoblasts

RNA (5 μg) isolated from osteosarcoma tumor tissue from three patients was purchased from OriGene Technologies (Rockville, MD). The Patient 1 RNA (CR559626) was isolated from a humerus parosteal osteosarcoma. Patient 2 RNA (CR562179) was isolated from a metastatic osteosarcoma. Patient 3 RNA (CR560767) was isolated from a recurrent osteosarcoma. Normal human osteoblast RNA was obtained from Cell Applications (Cat. #406-R10a) (San Diego, CA). Human osteoblast genomic DNA was obtained from ScienCell Research Laboratories (Cat. #4609) (Carlsbad, CA). Human osteosarcoma cell lines SaOS-2 (HTB-85) and U2OS (HTB-96) were obtained from the American Type Culture Collection (Manassas, VA).

### Cell Culture

SaOS-2 cells were grown in McCoy’s 5a Modified medium containing 15% fetal bovine serum (FBS) (Atlanta Biologicals, GA) and penicillin/streptomycin (50 I.U./50 μg per ml). The U2OS cells were grown in the same medium but with 10% FBS. Both cell lines were grown at 37°C in a 5% CO_2_ humidified cell culture incubator.

### RNA isolation, cDNA synthesis and qPCR

RNA was isolated from cell lines using the SV Total RNA Isolation System (Promega). 1 to 1.5 μg of total DNA was converted to cDNA using the QuantiTech Reverse Transcription Kit (Qiagen) or Maxima First Strand cDNA Synthesis Kit (Fermenta Life Science) according to the manufacturer’s instruction. The Maxima First Strand kit was used only for human osteosarcoma tissue RNA samples. Real time PCR (qPCR) was carried out using a StepOne Real Time PCR System (Applied Biosystems). The standard amplification conditions were 95°C for 15 seconds and 60°C for 1 minute for 40 cycles. Custom Taqman primer-probes were used for analysis of promoter A and B specific transcripts [32]. A dilution series of the purified and quantified *WNT5A* promoter A and promoter B PCR products were amplified along with the experimental cDNA samples to generate standard curves for quantification of transcript amounts. GAPDH transcripts were used as internal controls using GAPDH primer-probes (Applied Biosystems Hs99999905). Each specific primer-probe cDNA 10 μl reaction was run in triplicate prepared from a Master Mix of 33 μl containing 1.65 μl specific TaqMan primer-probe, 16.5 μl TaqMan Gene Expression Master Mix (Applied Biosystems) and 3.3 μl of cDNA sample (in some cases diluted 1:5). The unpaired Student’s t-test was used to determine significance between specific values.

### Transient transfection and luciferase assay

Osteosarcoma cancer cells SaOS-2 were grown to 80% confluency in 24-well plates at 2 x 10^4^ cells per well. Promoter A and promoter B luciferase constructs were individually transfected into the cells along with the *Renilla* control vector (phRL-SV40; Promega) using the NanoJuice Transfection Reagent (Novagen). Transfection was performed according to the manufacturer’s protocol. 48 hours after transfection, cells were collected. The medium was removed from each well and 500 μL of 1X Phosphate Buffered Saline (PBS) was added and removed. 150 μl of Passive Lysis Buffer (Promega) was added and the plate incubated for 15 minutes at room temperature on a shaker. The cell lysates were assayed for firefly and *Renilla* luciferase activity utilizing the Dual-Luciferase Reporter Assay System (Promega). 20–30 μl samples from each well were transferred to a 96 well black-walled plate. The samples were assayed on a Synergy 2 multimode microplate reader (BioTek). Pairwise comparisons were made between all samples for a given construct to determine significance (p<0.05) (ANOVA).

### Bisulfite sequencing

Genomic DNA was isolated from SaOS-2 and U2OS cells using the Genomic DNA Isolation Kit (Zymo Research). Osteoblast genomic DNA was purchased. 500–550 μg of genomic DNA was bisulfite converted using the EZ DNA Methylation Kit (Zymo Research) according to the manufacturer’s instructions. The bisulfite specific primers were used to amplify the CpG regions or portions of these regions. Typically 15–60 ng of converted DNA was amplified using ZymoTaq DNA polymerase (Zymo Research). Annealing temperatures were unique for each primer set (S1 Table). The other amplification parameters were identical: pre-incubation for 95°C for 10 min.; denaturation at 95°C for 30 sec; extension at 63°C for 30 sec. The PCR products were size verified, gel purified using the QIAQuick Gel Extraction Kit (Qiagen) and the purified PCR product subcloned into either the pCR^®^2.1-TOPO vector using the TOPO cloning kit (Invitrogen) or pGEM-T vector (Promega). After transformation into DH5α *E*. *coli* cells, colonies containing plasmids with inserts were verified. Plasmids were purified using the Plasmid Miniprep Kit (Promega). Sequencing of plasmid inserts was by Eurofin MWG Operon. Obtained sequences were aligned to a computer-generated sequence of the same region, assuming all CpG’s are methylated (hence C’s unconverted) and all other C’s are completely converted. The unpaired Student’s t-test was used to determine if the methylation difference was significant between two samples

### Treatment of cells with 5-azacytidine

SaOS-2 cells were plated in 10 cm plates at a density cell of 5 x 10^5^ cells and grown overnight. The cells were treated with 0 and 1 μM 5-azacytidine (5-aza) and grown for four days with one change of medium and re-addition of 5-aza. Cells were collected and RNA isolated from the cells as previously described. Promoter A and promoter B transcripts were quantified by qPCR as previously described.

#### ChIP Analysis

ChIP assays were performed using the ChIP-IT Express kit (Active Motif), according to their directions but with the following specific details. U2OS cells were grown to 70% confluency in nine 10 cm plates and fixed for 10 minutes at room temperature in the 37% formaldehyde with preservative (10–15% methanol). This provided enough material for three samples. Chromatin shearing of the fixed and washed cells was immediately conducted. Freezing of the fixed cells, prior to sonication, made it more difficult to shear the chromatin to the correct size. The U2OS fixed cells were sonicated using a 60 Sonic Dismembrator (Fisher Scientific) at 10 pulses of 20 seconds ON and 40 seconds OFF between levels 1 and 2. If needed, pulse numbers were adjusted, based on microscopic examination of the cells; disappearance of nuclei was found to correlate with sufficient sonication. Aliquots from each of the three samples were removed before storing at -80°C and DNA was purified and analyzed to determine chromatin size distribution. Sheared chromatin with DNA between 200–1500 base pair was used for immunoprecipitation. The concentration of DNA in the sheared chromatin was determined by reading the purified DNA sample at 260/280 optical density.

A 10 μl aliquot of the sheared chromatin was removed and saved as “Input DNA” prior to immunoprecipitation. 7 μg of sheared chromatin was used for each immunoprecipitation. Antibodies used for immunoprecipitation included RNA Polymerase II (Active Motif), rabbit anti-IgG (Active Motif), mouse anti-H3K27me3 (mAbcam ab6002), rabbit H3K9me3 (Abcam ab8898), and mouse anti-H3K4me3 (mAbcam ab1012). A bridging antibody (Active Motif 53017) was used with RNA polymerase II and the mouse monoclonal antibodies. The 100 μl reaction consisted of 25 μl of Protein G Magnetic Beads, ChIP Buffer 10 μl, 7 μg of sheared chromatin, 1 μl Protease Inhibitor Cocktail, 1–3 μg antibody, and as indicated, a bridging antibody, and brought to volume with water. Immunoprecipitation was performed overnight at 4°C after which the DNA was purified using the Chromatin IP DNA Purification Kit (Active Motif).

ChIP enrichment was determined by qPCR using SYBR-Green reagents. Reactions included each ChIP sample and INPUT DNA amplified with target primers. All reactions were run in triplicate. At least three different dilutions were made of the Input DNA to ensure linearity. GAPDH primers were also run with the ChIP samples as a standard, highly expressed gene. The ChIP-qPCR data was normalized using the Percent Input Method. Essentially, the Ct value of the Input was adjusted to 100% by converting the dilution factor of the Input to cycles (log_2_ of dilution factor) and subtracting the number from the obtained Input Ct value. The %INPUT value of each ChIP sample and IgG controls were calculated using the formula 100*2^[Adjusted input-Ct(IP)]. The significance of the %INPUT value of a particular ChIP sample compared to the %INPUT value of the control IgG was determined using the unpaired Student’s t-test to evaluate the enrichment to a given genomic region. Then, enrichment comparisons were made between regions.

## Results

### *WNT5A* promoter B activity is reduced in osteosarcoma cell lines and primary tumor tissue in comparison to normal osteoblasts

**Fig 2:**
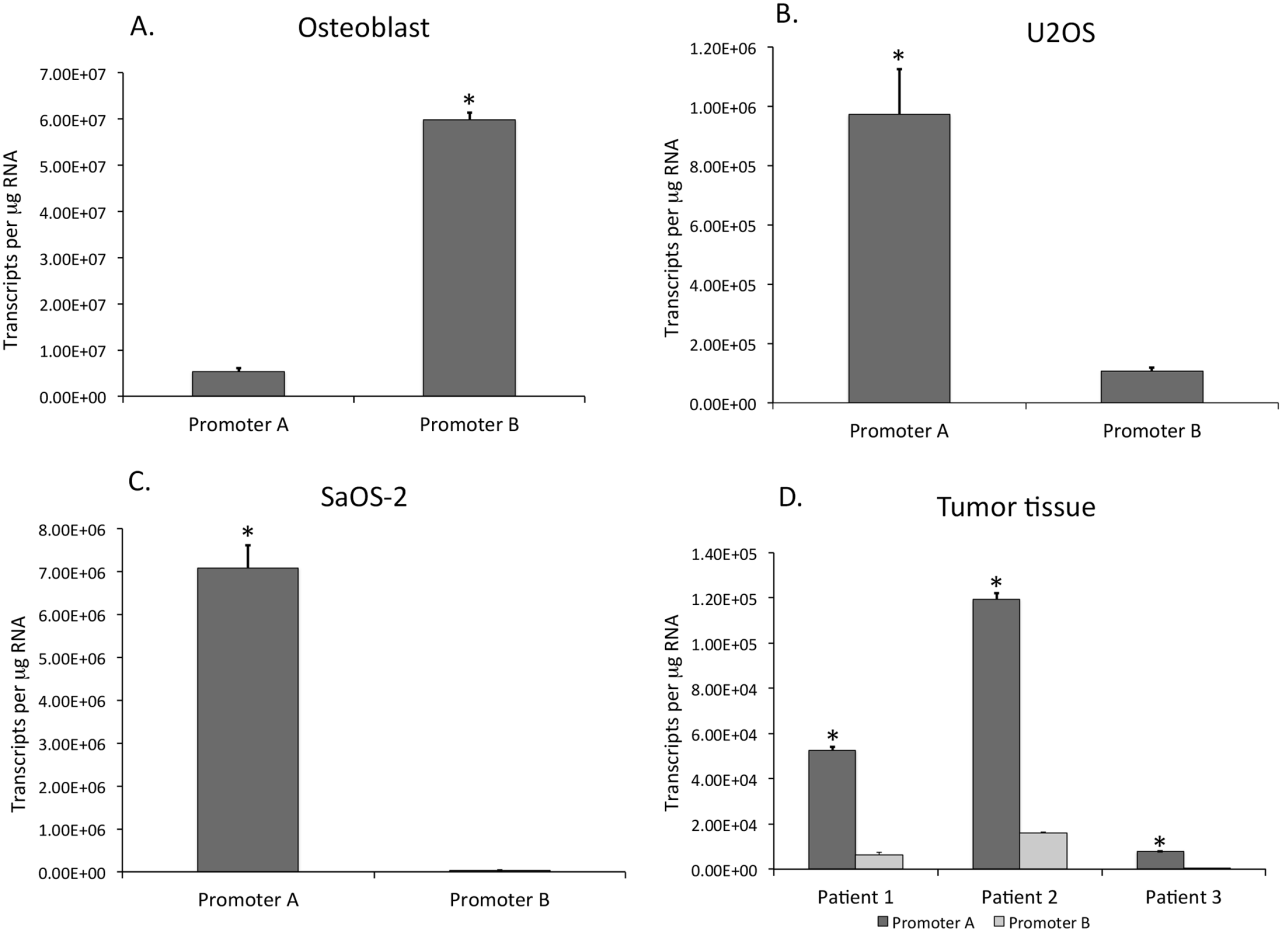
*WNT5A* promoter A and promoter B transcript levels in RNA isolated from A) normal human osteoblasts, B) U2OS and C) SaOS-2 human osteosarcoma cells lines, and D) osteosarcoma patient tumor tissue. Transcript copy numbers were quantified by qPCR using standard curves generated from purified promoter A and promoter B specific PCR products and expressed per μg RNA. Bars are plus/minus standard error of the mean (n = 3). Asterisk indicates promoter A and promoter B levels significantly different from one another at p<0.05 (unpaired Student’s t-test).

The levels of *WNT5A* promoters A and B transcripts were quantified in RNA from normal osteoblasts, osteosarcoma cell lines U2OS and SaOS-2, and osteosarcoma tumor tissue from three different individuals (Fig 2). In normal osteoblasts, both promoters A and B transcripts were detected, although numbers of promoter B transcripts were approximately 11-fold greater than those of promoter A (Fig 2A; S2 Table) and the A to B transcript ratio was 0.09 (S2 Table). In contrast, promoter B transcripts were reduced or nearly undetectable in osteosarcoma cell lines U2OS and SaOS-2 (Fig 2B and 2C). The A to B ratio in SaOS-2 cells was greater than 200 (S2 Table).

There were fewer promoter B transcripts than promoter A transcripts in the osteosarcoma cell line U2OS but on average the A to B ratio in U2OS cells was approximately 12 (S2 Table), suggesting that promoter B is more transcriptionally active in U2OS than in SaOS-2 cells. Together, the results in osteosarcoma cell lines and normal osteoblasts indicate that promoter B activity is reduced or inactivated during progression from normal to transformed osteosarcoma cells.

To determine if the reduction in promoter B transcript levels is a characteristic of osteosarcoma tumor tissue, we measured *WNT5A* promoter A and B transcript levels in osteosarcoma tissue from three individual patients. As shown, in all three tissue samples, promoter B transcript levels are lower than promoter A transcripts (Fig 2D). Patients 1 and 2 had A to B ratios of 8.7 and 7.5, respectively, whereas Patient 3 had a ratio of 78.5 (S2 Table). These data suggest that a reduction in promoter B transcripts is a feature of osteosarcomas.

### Transfected *WNT5A* promoters A and B reporter constructs are active in SaOS-2 cells

**Fig 3:**
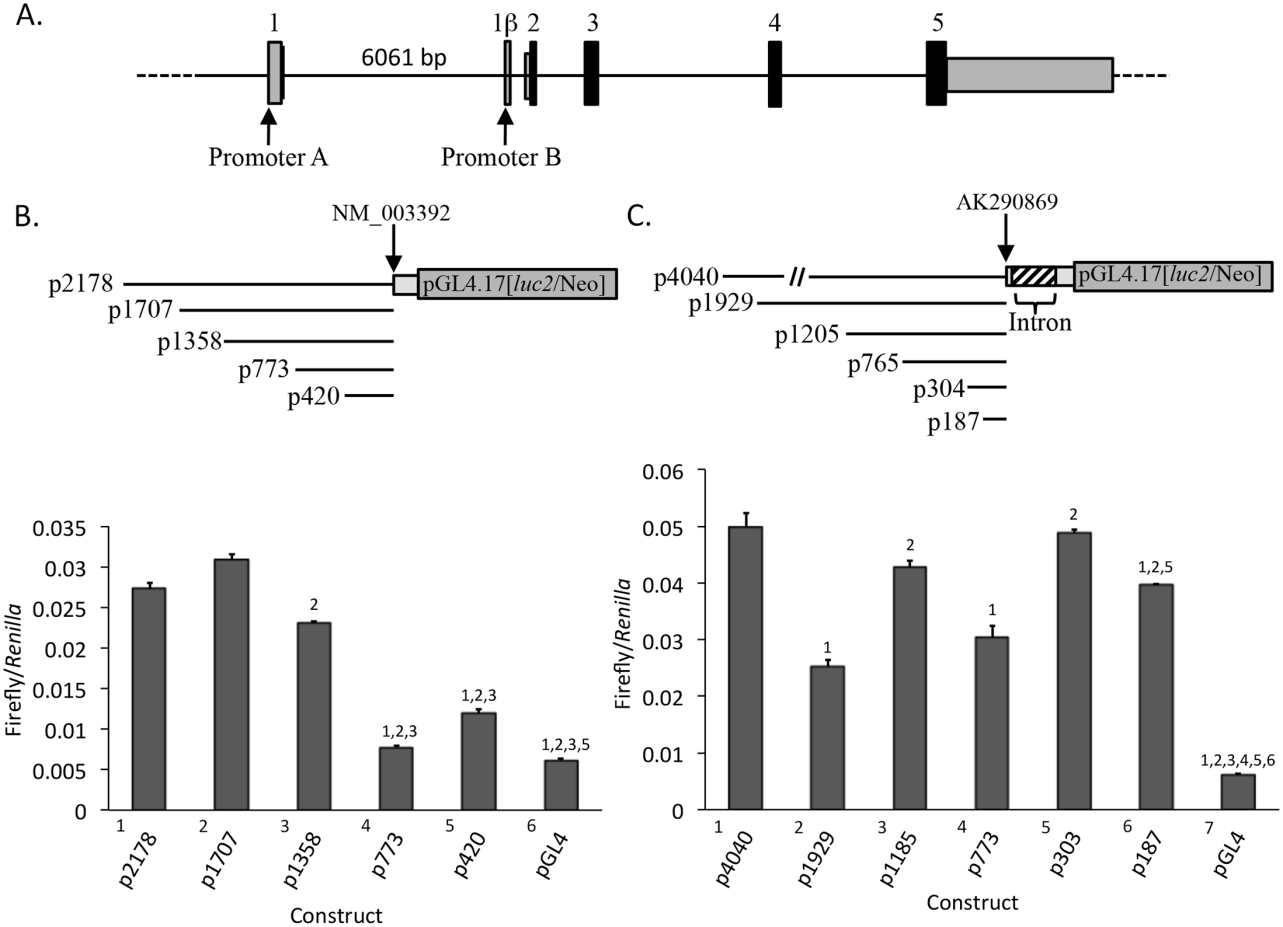
Promoter A and promoter B luciferase reporter constructs are expressed in SaOS-2 cells. A) Genomic region used for the construction of the luciferase reporter vectors. The numbered boxes are exon sequences. Black boxes are coding regions; gray boxes are noncoding regions. Lines between boxes are intron sequences. Cloned sequences of promoter A are upstream of exon 1. Promoter B cloned sequences include sequences downstream and upstream of exon 1β, located within the promoter A 6061 bp intron 1. B) Promoter A and C) promoter B reporter constructs used for transient transfection assays into SaOS-2 cells. The plasmid numbers are base pair upstream from the beginning of exon 1 (promoter A) as defined by the cDNA RefSeq NM_003392 or from exon 1β (promoter B) as defined by cDNA sequence AK290869. Below each set of constructs are the results of the transfection assays, expressed as firefly luciferase per *Renilla* control luciferase. Bars are plus/minus standard error of the mean, n = 4. Pairwise comparisons were made between all constructs (ANOVA). Only comparisons between a given construct and those longer than itself, showing a significant differences (p<0.05), are indicated (numbers above the bars). Constructs are numbered to the left, below the x-axis, along with their names.

One possibility for the lack of promoter B activity in osteosarcoma cells is the absence or reduced levels of transcription factors required for promoter B activity. We tested various promoter B luciferase reporter constructs for expression by transient transfection. For comparison we assayed promoter A luciferase reporter constructs. This analysis also provided for functional analysis of the promoter A and promoter B regulatory sequences in osteosarcoma cells. Promoter A and promoter B upstream sequences were obtained from the genomic region shown in Fig 3A and used to generated the luciferase reporter constructs shown in Fig 3B and 3C. These were transfected into SaOS-2 cells along with a *Renilla* reporter vector to control for transfection efficiency. Promoter A constructs containing 2178, 1707, and 1358 bp of upstream sequence expressed at a similar level, whereas constructs with only 773 and 420 bp expressed at a significantly reduced level (approximately one third to one half of the larger constructs) (Fig 3B). These results suggest the presence of an enhancer between 1358 and 773 bp for promoter A.

All of the promoter B constructs, including the 187 bp construct, are expressed in SaOS-2 cells, indicating that the necessary transcriptional components are available for driving expression from the included sequences (Fig 3C). There is some variation in expression levels between constructs, suggesting the presence of regulatory sequence elements, however no clear pattern emerges and the differences are relatively small. In general, these data indicate that for *WNT5A* promoter B, 187 bp of upstream sequence is sufficient for a level of expression comparable to a construct with 4040 bp of upstream sequence, and that no major enhancers are located in the upstream sequences.

### Specific *WNT5A* promoter B CpG enriched sequences are methylated in SaOS-2 and U2OS cell lines

**Fig 4:**
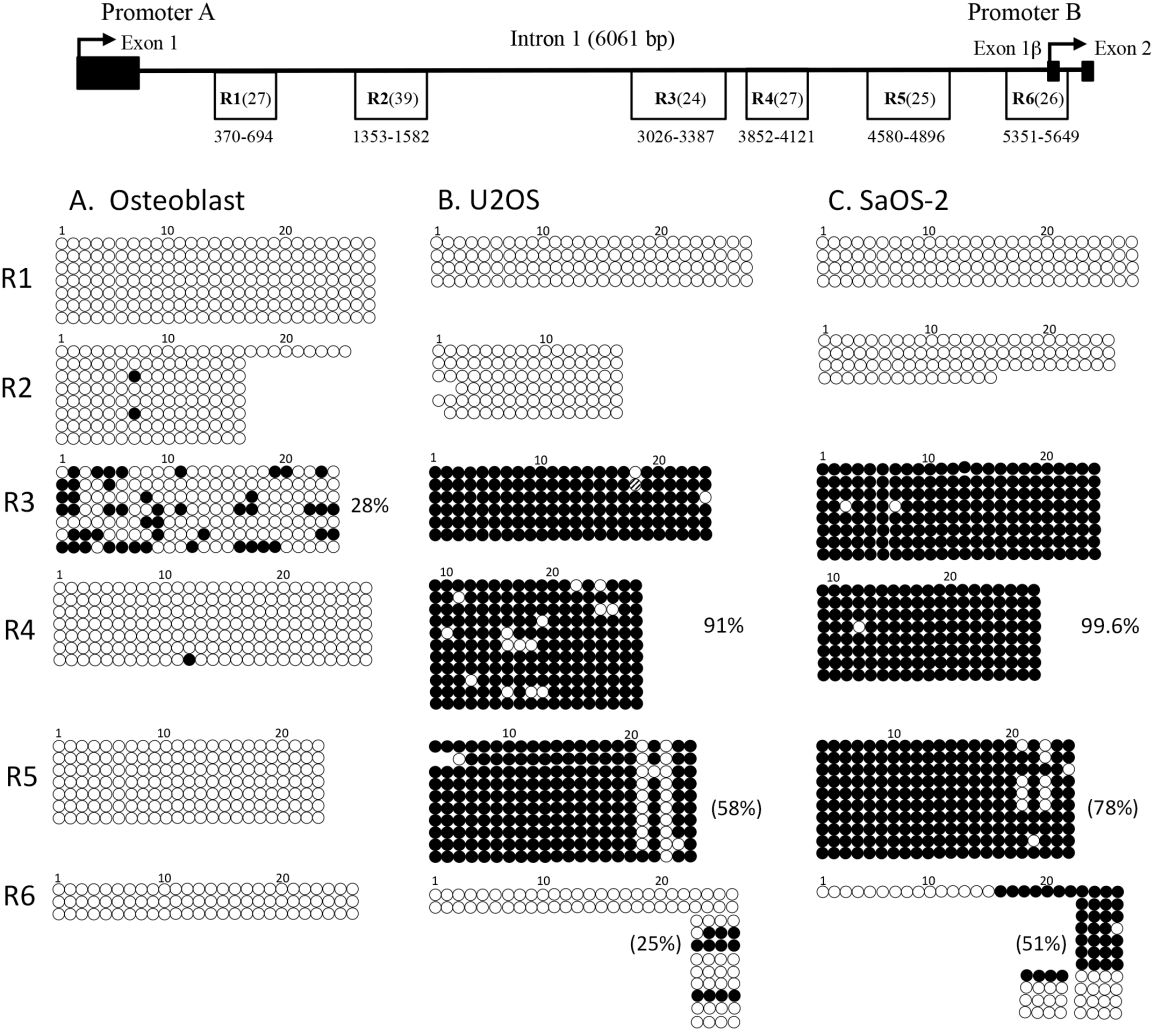
Similar pattern of CpG island methylation in the *WNT5A* intron 1 of SaOS-2 and U2OS osteosarcoma cell lines. Shown at the top are the CpG enriched region (R1–R6) locations in *WNT5A* intron 1. The numbers indicate the relative base pair location within the intron sequence with the first base pair of intron 1 designated as number 1. The total numbers of CpG’s per region are given in parenthesis. Bisulfite sequencing analysis for CpG region R1–R6 for A) normal osteoblasts, B) U2OS and C) SaOS-2 osteosarcoma cell lines. Closed circle = methylated CpG; open circle = unmethylated CpG. Each row is an individual sequenced clone. Only partial clones could be obtained for some regions. The percent methylated for region R6 CpG’s 23–26 and region R5 CpG’s 21–25 based on the clones shown for U2OS and SaOS-2 are given in parenthesis. The percent methtylated for the entire region R4 is shown for both U2OS and SaOS-2 and region R3 for osteoblast.

It is likely that lack of promoter B expression is due to DNA methylation, considering that promoter A associated CpG’s have been found to be methylated in various cancers (13, 14, 15, 16, 17, 18, 30). We choose to focus on CpG’s in the entire 6061 bp intron 1 (part of the promoter A primary transcription unit; see Fig 1) as these sites have the potential to influence promoter B transcription. We compared the University of California Santa Cruz (UCSC) Genome Brower CpG island map, NCBI Epigenomic Browser CpG Island map, and the results from “Emboss CpG” plot and selected six CpG sequence regions referred to as regions R1 to R6 (Fig 4). These sequences were analyzed by bisulfite sequencing in normal osteoblast and U2OS and SaOS-2 osteosarcoma cell DNA (Fig 4A–4C).

The CpG regions R1, R2, R4, R5 and R6 in the osteoblast DNA were essentially unmethylated, whereas region R3 was approximately 28% methylated (Fig 4A). In the U2OS and SaOS-2 cells, regions R1 and R2, which are further upstream and more proximal to promoter A, were completely unmethylated (Fig 4B and 4C). In contrast, regions R3, R4 and R5 were highly methylated in both U2OS and SaOS-2 cells (Fig 4B and 4C). Region 3 is approximately 99% methylated for both U2OS and SaOS-2 cells. U2OS region R4 clones were approximately 95.1% methylated in comparison to 99.6% methylated for SaOS-2 region R4 and this difference is significance (p<0.003). Region R5 in U2OS is approximately 91% methylated at all CpG’s for the 10 clones analyzed but displays a group of unmethylated CpG’s that cluster at 21, 22, 23, and 24 with the majority at positions 21 and 23. SaOS-2 region R5 shows a similar pattern of unmethylated CpG’s in region R5. In comparison, the percent methylation in region R5 CpG’s 21–25 is 58% for U2OS cells and 78% for SaOS-2 cells but this difference does not reach significance (p<0.0528). One possibility for this pattern is interference of de novo methylation at these CpG’s due to protein binding, possibility by a transcription factor. These comparisons suggest that methylation is increasing from U2OS to SaOS-2 cells.

CpG region R6 is associated with the promoter B start of transcription and exon1β, possessing 26 CpG’s. In osteoblasts, three full length region R6 clones were unmethylated (Fig 4A). In U2OS cells, the two full length clones obtained were unmethylated (Fig 4B). Of the one full-length SaOS-2 clone obtained, only the last 11 CpG’s were methylated (Fig 4C). A different primer set was used to amplify a sub-region of region R6 containing only four of the CpGs. 25% of these four CpG’s were methylated in U2OS cells. In contrast, in SaOS-2 cells, based on 15 clones, 51% of these four CpG’s were methylated. This difference was significant (p<0.0009).

The increase in methylation of sub-region 6 in SaOS-2 cells is associated with an approximately 30-fold reduction in promoter B transcript numbers in comparison to U2OS cells (calculated from transcript numbers in S2 Table). There is the possibly that methylation of other upstream CpG islands (regions 3, 4, and 5) contribute to the reduced transcription of promoter B, based on the finding that normal osteoblast cells have approximately 500X more promoter B transcripts than U2OS cells (S2 Table). Sub-region 6 is unmethylated in osteoblasts and only 25% methylated in U2OS cells.

### Re-activation of promoter B in SaOS-2 cells with 5-azacytidine

**Fig 5:**
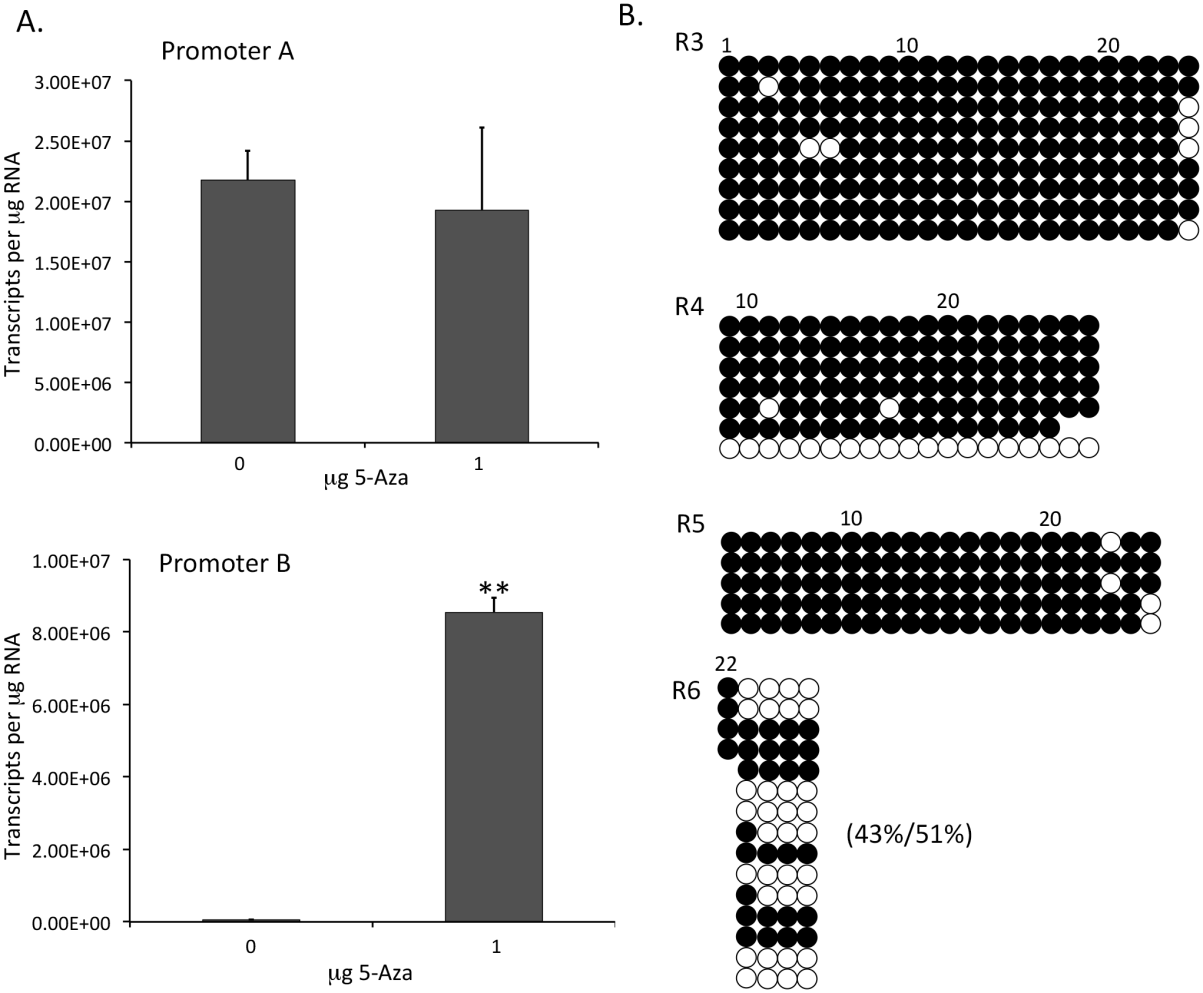
Promoter B is reactivated in SaOS-2 cells by treatment with 5-azacytidine (5-aza). A) SaOS-2 cells were treated with 0 and 1 μM 5-aza for 4 days. Promoter A and promoter B specific transcripts were quantified by qPCR using standard curves. Bars are plus/minus standard error of the mean, n = 4, **p<0.001. B) Bisulfite sequencing was completed for CpG regions R3 to R6 using DNA isolated from SaOS-2 cells treated with 1 μM 5-aza for 4 days. The percent methylated for region R6 comparing 5-aza treated to untreated (from Fig 4) is shown in parenthesis.

Next we wanted to determine if promoter B could be re-activated by de methylation to confirm that DNA methylation has a role in promoter B inactivation in SaOS-2 cells. SaOS-2 cells were treated with 0 and 1 μM of 5-azacytidine (5-aza), a DNA methyltransferase inhibitor, for 4 days. RNA was purified and relative levels of promoter A and promoter B transcripts determined. 5-aza had essentially no effect on levels of promoter A transcripts, as promoter A is active in SaOS-2 cells (Fig 5A). At 1 μM 5-aza, there was a 120-fold increase in promoter B transcripts over the untreated controls (Fig 5A).

To determine in what manner the 5-aza is altering the pattern of DNA methylation in CpG regions R3, R4, R5 and R6, previously shown to be methylated, we completed bisulfite sequencing using DNA from SaOS-2 cells treated for 4 days with 1 μM 5-aza (Fig 5B). Again, we were unable to amplify the entire region R6, so only a sub-region including CpG’s 23 to 26 was analyzed. As shown, 5-aza had a slight effect on sub-region R6; de-methylation was approximately 8% compared to untreated SaOS-2 cells (Fig 5B) but this difference didn’t reach significance (p<0.465). There were no significant changes in methylation for regions R5, R4, and R3, although one completely non methylated clone was isolated for region R4.

### Decreased enrichment of the active histone mark H3K4me3 at promoter B in comparison to promoter A in U2OS and SaOS-2 cells

**Fig 6:**
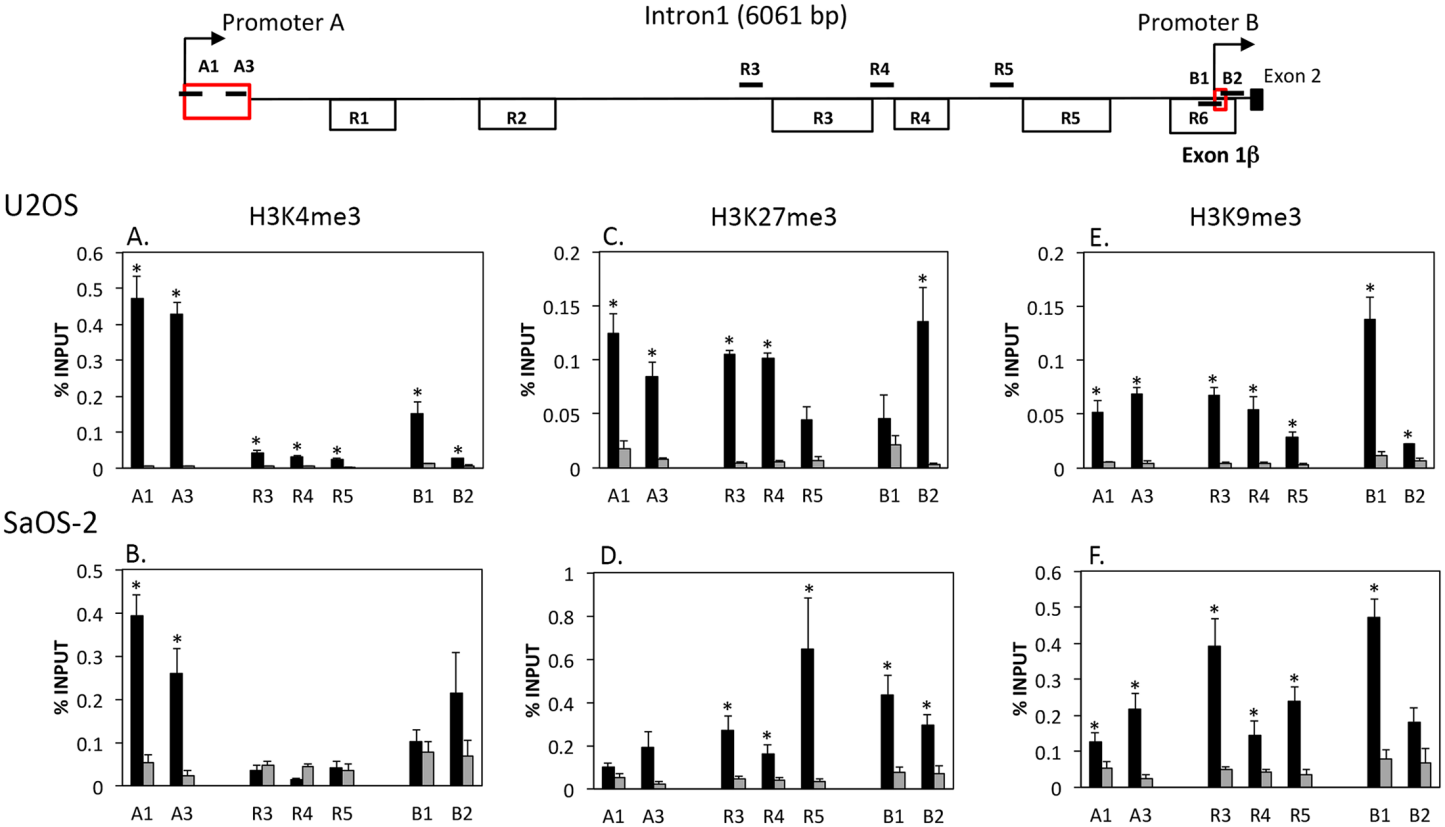
Histone modifications in *WNT5A* promoter A, promoter B, and intron 1 of U2OS and SaOS-2 cells. A) Map of the *WNT5A* intron 1 genomic region showing the locations of the promoter A and promoter B ChIP primers (dark bars above line except for B1, which is below the line) relative to the CpG regions (R1–R6) and Exon 1 (promoter A) and Exon 1β (promoter B), shown as red boxes downstream of the arrows. B) Chromatin prepared from U2OS and SaOS-2 cells was analyzed by chromatin immunoprecipitation (ChIP) using antibodies against H3K27me3, H3K4me3, and H3K9me3 and IgG as a control. The % INPUT was determined by qPCR as described in the Materials and Methods. Black columns represent the averages of 6 to 12 values for SaOS-2 and 3 to 6 values for U2OS. Gray bars are the averages for the IgG control values. Bars are standard error of the mean. * indicates comparisons of the anti-histone antibody to IgG control values that are significantly different (p<0.05).

Genomic patterns of DNA methylation affecting promoter activity in both normal and cancer cells are associated with specific histone modifications [35, 36, 37, 38]. As sub-region R6 is only partially methylated, histone modifications could contribute to the down regulation in promoter B activity. We used ChIP analysis to analyze both promoters A and B regions for repressive marks H3K27me3 and H3K9m3 and the active mark H3K4me3 in U2OS and SaOS-2 cells (Fig 6). In U2OS cells, the background %INPUT values from the IgG ChIP reactions were generally lower than that measured in SaOS-2 cells, although this did not appear to impact the pattern of enrichment across the region.

H3K4me3 was detected at all regions in U2OS cells (Fig 6A). However, %INPUT values were significantly higher (p<0.05) at probes A1 and A3 (promoter A) than R3, R4, R5, and B1 and B2 (promoter B). A1% INPUT was 3 to 17-fold greater than B1 and B2, respectively. Although the pattern was very similar in SaOS-2 cells, only the A1 and A3 probes were significantly different (p<0.05) from the IgG controls (Fig 6B). Values for %INPUT were similar for the A1 and A3 probes (promoter A) for both SaOS-2 and U2OS in the range of 0.3 to 0.55. These results suggests that H3K4me3 is more enriched at promoter A but reduced at promoter B in both U2OS and SaOS-2 cells.

The repressive histone mark H3K27me3 shows similar but low levels of %INPUT (less then 0.2) for U2OS cells, including both promoters A and B exons 1 and 1β, respectively, and the non-promoter associated CpG island regions R3, R4, and R5 (Fig 6C). In SaOS-2 cells, there is increased enrichment of H3K27me3 in association with the non-promoter regions R3, R4, and R5 and the promoter B region (probes B1 and B2), whereas association with the A1 and A3 probes (promoter A) is not significant (Fig 6D). Similarly, although the %INPUT values for the repressive histone mark H3K9me3 were significant for all probes for U2OS cells, the values were less than 0.1 with the exception of probe B1 (promoter B) (Fig 6E). Probe B1 had an average % INPUT of 0.14. The H3K9me3 pattern is similar for SaOS-2 cells except that overall % INPUT values are higher across the region and the value for R3 is proportionally higher (Fig 6F). Assays using the GAPDH primers gave the expected results of high enrichment for RNA pol II and H3K4me3 (data not shown).

## Discussion

Our results show that the *WNT5A* alternative promoters A and B are differentially expressed in both osteosarcoma cell lines SaOS-2 and U2OS and patient osteosarcoma tumor tissues. In normal osteoblasts, promoter B transcripts are nearly 11X greater than promoter A transcripts, whereas in osteosarcoma cells promoter B transcripts are reduced or barely detectable. The decrease in promoter B activity is associated with increased DNA methylation of specific CpG enriched sequences and reduced enrichment of the active histone mark H3K4me3.

Methylation of CpG island sub-region R6, located in promoter B exon 1β, and promoter B transcript levels display an inverse relationship. Osteoblasts showed no methylation at any CpG’s in sub-region R6 and had the highest number of promoter B transcripts: approximately 500X more than U2OS and 1700X more for SaOS-2 cells (values derived from S2 Table). SaOS-2 cells had 51% methylation in sub-region R6 and the lowest number of transcripts. U2OS cells with 25% methylation had over 3X more promoter B transcripts than SaOS-2 cells. In general, our analysis suggests that even the relatively low level of sub-region R6 methylation in U2OS cells leads to reduced promoter B activity.

Both U2OS and SaOS-2 cells displayed high levels of methylation for CpG island regions R3, R4, and R5. In SaOS-2 cells region R4 is significantly more methylated. Region R5 CpG’s 21 to 24 in both U2OS and SaOS-2 cells display a distinct pattern of methylation with CpG’s 21 and 23 tending to be unmethylated. It is possible that these particular CpG’s are protected from methylation. Analysis of this region for transcription factor binding sites reveals those for AP-2, WT-1, and c-Jun that are specifically associated with the sequence region including CpG’s 21–25 but not in other region R5 sequences (data not shown).

Treatment of SaOS-2 cells with 5-aza led to a large increase (120X) in promoter B activity, supporting the conclusion that DNA methylation is primarily responsible for the decrease in promoter B activity in this cell line, although transcript levels did not reach those of normal osteoblasts. It is surprising that only a slight and insignificant decrease in methylated CpG’s was measured in sub-region R6 of the 5-aza treated cells, as we predicted that de-methylation of the CpG’s in this region would have the greatest impact on promoter activity. Initially, we had difficulty cloning the entire region R6 and found it necessary to use a different probe set. Should it be possible to get full length R6 clones and more of them, we would predict that there would be significance in the percent change in the R6 region. No other regions showed a significant decrease in methylation.

Our analysis of promoter B upstream sequences by transient transfection into SaOS-2 cells supports the conclusion that all the factors required for promoter B expression are available. This indicates that promoter B activation by 5-aza is not due to secondary effects on other genes. Moreover, no enhancer sequences upstream of 187 bp were defined. In fact, 187 bp appears to be sufficient for a high level of expression. This suggests that the DNA methylation status of regions R3, R4, and R5 will have little or no impact on promoter B activity unless the methylation in these regions is affecting chromatin structure in a more general manner that is unrelated to specific gene regulatory sequences, and that these changes are impacting promoter B. Apparently, this is not the case, as 5-aza treatment increased promoter B activity but did not alter methylation in these CpG regions.

Our results also suggest that reduced promoter B activity in osteosarcoma cell lines is due to a combination of DNA methylation and histone modifications.

**Fig 7:**
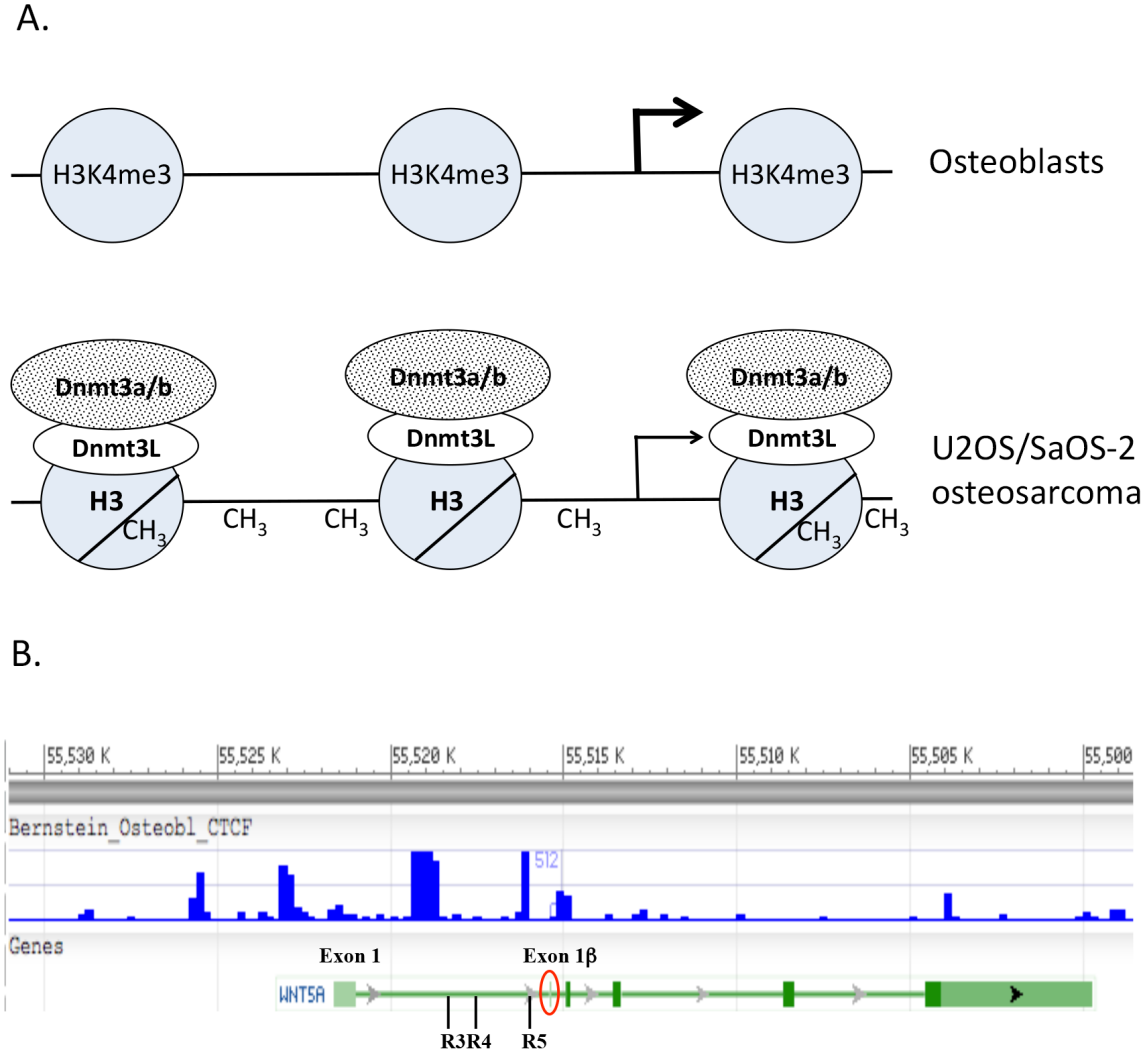
Models for changes in DNA methylation of the *WNT5A* promoter B region during progression of osteoblast cells to osteosarcoma. A) Presumed enrichment of H3K4me3 in the promoter B region of normal osteoblast would block binding of the DNA methyltransferase enzyme complex, keeping the DNA unmethylated. In osteosarcoma cells, the level of H3K4me3 decreases in the promoter B region R6 and unmethylated H3 increases, allowing the Dnmt3L/Dnmt3a/b complex to bind to the H3 and *de novo* methylate the DNA, leading to reduced transcription of promoter B. B) NCBI Epigenomics search for CTCF binding in the *WNT5A* genomic region of human osteoblasts. Location of the CpG regions R3, R4, and R5 are indicated, along with promoter A exon 1 (box) and promoter B exon 1β (box at arrow). Reduced CTCF binding in osteosarcoma cells could lead to increased methylation of associated DNA regions.

The active histone mark H3K4me3 was more enriched in promoter A then promoter B. H3K4me3 is generally associated with active promoters and occurs in regions of unmethylated DNA [39]. The reduction in H3K4me3 at promoter B could, in part, explain the decrease in promoter B activity in U2OS and SaOS-2 cells. Lack of methylated H3K4me3 at gene promoters allows binding of Dnmt3L and Dnmt3a/b and *de novo* DNA methylation, whereas the H3K4me3 form appears to block de novo methylation [40, 41]. As such, the reduction in H3K4me3 enrichment at promoter B would lead to increased DNA methylation by Dnmt3L/Dnmt3a/b activity and reduced promoter B activity (Fig 7A). The fact that CpG region R6 of U2OS and SaOS-2 cells are only partially methylated indicates that sufficient H3K4me3 may still be present to block some methylation. Increased enrichment of the repressive histone H3K27me3 across the R3, R4, R5 and promoter B regions of SaOS-2 is also likely contributing to an altered chromatin structure and reduced promoter B expression, in comparison to U2OS cells. ChIP analysis of normal osteoblast cells should provide confirmation of this model.

Cancer cells tend to show general global hypo-methylation but hyper-methylation of CpG islands, particularly those associated with promoters [42]. CpG island regions R3, R4 and R5 are methylated in both SaOS-2 and U2OS cells. Region R3 shows 28% methylation in normal osteoblasts, indicating that these sequences may be sites of normal genomic methylation. Aberrant methylation in cancer could result from stochastic effects, where DNA methylation takes place randomly in retrotransposon sequences such as LINE (Long Interspersed Elements) and SINE (Short interspersed Elements), which are present throughout the genome [43, 44]. Analysis of the intron 1 sequences, however, using the UCSC Genome Browser shows that there are no such repeats associated with intron 1.

Another possibility for the methylation pattern could be due to insulator proteins, e.g. CTCF and USF 1/2. Lack of CTCF has been associated with increased DNA methylation [45, 46]. In particular, mutated CTCF binding sites in the Rb promoter lead to progressive DNA methylation of the region [45]. An ENCODE search for these insulator protein binding sites in osteoblasts showed that *WNT5A* intron 1 includes binding sites for the insulator protein CTCF, which flank CpG regions R3, R4 and R5 and are also just downstream of exon1β (Fig 7B). A reduction in CTCF proteins in the U2OS cells may result in enhanced methylation of CpG regions R3, R4 and R5 and promoter B CpG region R6 sequences, leading to reduced promoter B transcription. In contrast, CpG regions R1 and R2 were not methylated in normal osteoblasts or the osteosarcoma cell lines. The more active promoter A in these cells is may influence regions R1 and R2, keeping them free of methylation.

Our study demonstrates that a reduction in promoter B activity occurs in osteosarcoma cancers, supporting the possibility that WNT5A isoform B is functioning as a tumor suppressor in this cancer. Bauer et al. [31] examined the WNT5A isoform transcript levels in three cancer cell lines and found that expression was variable. Hela cells expressed high levels of the B isoform (WNT5A-S in their study), whereas MDA-MB-231 (breast cancer) and SH-SY5Y (neuroblastoma) expressed lower levels of B relative to the A isoform (WNT5A-L in their study). As previously mentioned, they provided evidence that isoform B (WNT5A-S) enhanced cell proliferation, whereas isoform A (WNT5A-L) inhibited proliferation. Based on these results, the expectation is that cancer cells should express higher levels of isoform B transcripts than isoform A transcripts, contrary to what we found in osteosarcoma cells and to what Bauer et al. detected for two of their cancer cell lines. Clearly, there is a need for further analysis of the functional distinctions between the WNT5A isoforms. We are currently analyzing lines of SaOS-2 cells that overexpress isoform B.

## Supporting Information

S1 Table Bisulfite sequencing and ChIP primers and amplification conditions. Primer sequences used for amplification of sequences for bisulfite sequencing analysis and for ChIP analysis. Only the specific annealing temperatures are included.
(PDF)

S2 Table *WNT5A* promoter A and promoter B transcript numbers and A/B ratios. The transcript numbers determined from the analyses in Fig 2 were used to calculate the ratio of promoter A to promoter B transcripts (A/B) in the indicated samples.
(PDF)
