# Supplementary material for: Inactivation of the WNT5A Alternative Promoter B Is Associated with DNA Methylation and Histone Modification in Osteosarcoma Cell Lines U2OS and SaOS-2
Source: PLoS One. 2016 Mar 15;11(3):e0151392. doi: 10.1371/journal.pone.0151392 (PMC4792504; doi:10.1371/journal.pone.0151392)
Supplement: S1 Table — Primer sequences used for amplification of sequences for bisulfite sequencing analysis and for ChIP analysis. Only the specific annealing temperatures are included. (PDF) [file pone.0151392.s001.pdf]

Table 1. Bisulfite Sequencing and ChIP Primers and Amplification Conditions

Bisulfite Primers	Sequence (5'-3')	Anneal Temp	Size (bp)
W5AR1F	AGTAATAGGATAATGATTTAATTTAATAAA	45°C	393
W5AR1R	CAAAATACCTAAACTCACCCAATA		
W5AR2F	AGTTTTTAGATAGATTTTGTTAGGGGAGT	45°C	255
W5AR2R	CATAATACTAACCCCTTAAACACC		
W5APrBR2HF	GTTTAAGAGTTTTTTGTTTTTTTT		335
W5APrBR2HR	AAATACCAACCCTATATCCAACTAAC		
W5aR3F#2	GAGGATTTTTGTTTGGTTTTTTTATT	55°C	441
W5AR3R	AAACTAAATATTTTCCAACACTTTC		
W5AR4F	GTTTTGGGAATTGGTGGATTT	45°C	329
W5AR4R	AACCCAAACTACAAAATCAACTCTC		
W5AR4F3H2	GAATTTGTTATTAATTTTTTTGTTGTTTG	58°C	226
W5AR4R3H2	TACAAAATCAACTCTCCCCAAAA		
W5APrBR5nF	AGGGTTGTTTAGGTTTTTTTTGTTT	45°C	578
W5APrBR5nR	AACTCCAATTTCCCTTAATCCTAAT		
W5AR6F	TTTTTTTTAGTTTGGTTGAAGAA	55°C	393
W5AR6R	CCTAAATAATTCACTCAAAAACCC		
W5AR6H2F5	AATTAATTTTGGTTTTATTTGTTGTT	55°C	96
W5AR6H2R5	TCAATCCCTCCTAAATAATTCACTC		
**ChIP Primers**			
ChPrB1F	CCACCCCGCCTCCTTGG	60°C	127
ChPrB1R	GCCACCCTCCGTCCTCTCC		
ChPrB2F	CTCCACTCGCCTCCGTG	60°C	189
ChPrB2F	GCCTCCTTCCTGCTCGCTC		
ChPrA1F	GCCTCTCCGTGGAACAGTTGC	60°C	110
ChPrA1R	GATGCGCCCAGGAATGG		
ChPrA3F	CGCCAGTGCCCGCTTCAG	60°C	123
ChPrA3R	CAGCCGAGGAATCCGAGC		
ChR3F	TGGGTCAAATGGGCTTCTTCC	60°C	68
ChR3R	TCTGGGAGTGAAACGATGAGGG		
ChR4F	GAGATGCGACTCGTGAGGCTC	60°C	72
ChR4R	GCTCCAGGGTAGAGGTAGGCAG		
ChR5F	GGCGGCTCACGGAGAAAAAC	60°C	84
ChR5R	AAAAGGGGCAGAGGGGACC		
