# Supplementary material for: Inactivation of the WNT5A Alternative Promoter B Is Associated with DNA Methylation and Histone Modification in Osteosarcoma Cell Lines U2OS and SaOS-2
Source: PLoS One. 2016 Mar 15;11(3):e0151392. doi: 10.1371/journal.pone.0151392 (PMC4792504; doi:10.1371/journal.pone.0151392)
Supplement: S2 Table — The transcript numbers determined from the analyses in Fig 2 were used to calculate the ratio of promoter A to promoter B transcripts (A/B) in the indicated samples. (PDF) [file pone.0151392.s002.pdf]

Table 2 *WNT5A* Promoters A and B Transcript Numbers

	**Transcripts/μg RNA[1]**	**A/B Ratio[2]**
**Normal osteoblasts**	A – $5.3 \times 10^6 \pm 0.8 \times 10^6$<br>B – $5.9 \times 10^7 \pm 1.6 \times 10^6$	0.09 ±0.02
**Osteosarcoma cell lines**		
SaOS-2	A – $6.8 \times 10^6 \pm 0.5 \times 10^6$<br>B – $3.3 \times 10^4 \pm 0.7 \times 10^4$	224.5 ±65.2
U2OS	A – $9.7 \times 10^5 \pm 1.5 \times 10^5$<br>B – $1.1 \times 10^5 \pm 1.3 \times 10^4$	11.9 ±4.1
**Osteosarcoma tumor tissue**		
Patient 1	A – $5.3 \times 10^4 \pm 0.2 \times 10^4$<br>B – $6.3 \times 10^3 \pm 1.2 \times 10^3$	9.2 ±3.5
Patient 2	A – $1.2 \times 10^5 \pm 0.03 \times 10^5$<br>B – $1.6 \times 10^4 \pm 0.05 \times 10^4$	7.5 ±0.7
Patient 3	A – $7.9 \times 10^3 \pm 0.1 \times 10^3$<br>B – $1.1 \times 10^2 \pm 0.2 \times 10^2$	78.5 ±27.8

[1,2]± standard error; n= 3 or 6
